# Gene Loss Dominates As a Source of Genetic Variation within Clonal Pathogenic Bacterial Species

**DOI:** 10.1093/gbe/evv135

**Published:** 2015-07-10

**Authors:** Evgeni Bolotin, Ruth Hershberg

**Affiliations:** Rachel & Menachem Mendelovitch Evolutionary Processes of Mutation & Natural Selection Research Laboratory, Department of Genetics and Developmental Biology, The Ruth and Bruce Rappaport Faculty of Medicine, Technion-Israel Institute of Technology, Haifa, Israel

**Keywords:** bacterial evolution, gene loss, pangenome, clonal pathogens, sources of variation

## Abstract

Some of the most dangerous pathogens such as *Mycobacterium tuberculosis* and *Yersinia pestis* evolve clonally*.* This means that little or no recombination occurs between strains belonging to these species. Paradoxically, although different members of these species show extreme sequence similarity of orthologous genes, some show considerable intraspecies phenotypic variation, the source of which remains elusive. To examine the possible sources of phenotypic variation within clonal pathogenic bacterial species, we carried out an extensive genomic and pan-genomic analysis of the sources of genetic variation available to a large collection of clonal and nonclonal pathogenic bacterial species. We show that while nonclonal species diversify through a combination of changes to gene sequences, gene loss and gene gain, gene loss completely dominates as a source of genetic variation within clonal species. Indeed, gene loss is so prevalent within clonal species as to lead to levels of gene content variation comparable to those found in some nonclonal species that are much more diverged in their gene sequences and that acquire a substantial number of genes horizontally. Gene loss therefore needs to be taken into account as a potential dominant source of phenotypic variation within clonal bacterial species.

## Introduction

Bacteria can evolve by changes to the sequences of existing genes and regulatory regions and by changes to their gene content. Changes to gene content can occur through the loss or gain of gene functions (Snel et al. 2002; Jain et al. 2003). For convenience sake, we will from now on refer to the loss of gene function as “gene loss,” and to the gain of gene function as “gene gain.”

**Fig. 1.—:**
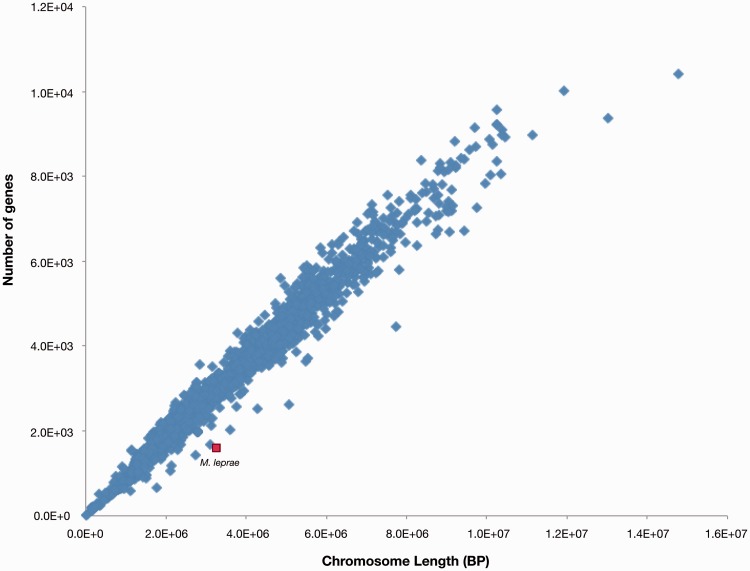
Genome size correlates very well with gene number, across bacterial genomes. Each dot within this graph represents a single bacterial genome. *Mycobacterium leprae* is highlighted as a clear outlier to this trend. The genome of *M. leprae* is very large relative to its functional gene count, due to uncharacteristically high maintenance of pseudogenes.

When a gene function is lost, the DNA of the gene in question can either be completely removed from the genome or be maintained in a deactivated from, referred to as a pseudogene. In most bacterial species, pseudogenes tend to be rapidly removed from the genome (Lerat and Ochman 2005; Kuo and Ochman 2010). As a result, most bacterial genomes contain only relatively small numbers of pseudogenes. Fitting with this, across bacterial genomes there is a very strong correlation between genome size and the number of functional genes encoded within the genome (*P* ≪ 0.001, according to a Spearman Rank correlation test; fig. 1). At the same time, exceptions exist, and some bacteria maintain many more pseudogenes than average. The most well-known example of such an exception is the bacterium *Mycobacterium leprae* (Gomez-Valero et al. 2007).

Gene gain is often achieved through the process of horizontal gene transfer (HGT) (Jain et al. 2002, 2003). HGT allows bacteria to acquire novel genes from other bacteria with which they cohabitate. Stable integration of the acquired DNA into the host’s genome requires recombination between endogenous and exogenous genetic material. Homologous recombination between related genes may lead to changes in existing gene sequences (Thomas and Nielsen 2005). At the same time, nonhomologous recombination may cause changes in the gene content of the host (Thomas and Nielsen 2005). The extent to which bacteria recombine and undergo HGT varies greatly between different bacterial species. Although some bacterial species recombine frequently, other bacterial species are highly clonal, evolving mainly through alteration of vertically inherited genes.

Highly clonal bacterial species include some important human pathogens, such as *Yersenia pestis* (causative agent of bubonic plague) (Achtman et al. 1999) and the *Mycobacterium tuberculosis* complex (MTBC) (causative agent of tuberculosis) (Hershberg et al. 2008; Comas and Gagneux 2011; Namouchi et al. 2012). Some of these clonal pathogens present a rather puzzling discrepancy, showing much phenotypic diversity between strains, despite near identity in gene sequences. The MTBC is a striking example of this discrepancy. A vast number of experiments and clinical observations show that there is much phenotypic diversity between MTBC strains in terms of virulence and immunogenicity (as reviewed in Coscolla and Gagneux [2010]). Seemingly in contrast to this extensive phenotypic diversity, sequence diversity within the MTBC is extremely low (Smith et al. 2006; Hershberg et al. 2008; Comas and Gagneux 2011; Namouchi et al. 2012). Orthologous genes are on average more than 99.6% identical in their amino acid sequences between different MTBC strains (see Results). It has previously been shown that a relatively high proportion of sequence differences between MTBC strains within orthologous genes carry functional consequences (Hershberg et al. 2008). Still, such sequence differences remain extremely rare. In addition, being a clonal pathogen, MTBC seems to undergo little to no HGT (Smith et al. 2006; Hershberg et al. 2008; Comas and Gagneux 2011; Namouchi et al. 2012), meaning that the ability of a given *M. tuberculosis* strain to acquire genes from external sources is quite limited. This raises the question of how phenotypic diversity is generated within the species with such high sequence similarity of orthologous genes and such low rates of recombination. We show that changes to gene content achieved through gene loss provide a major source of genetic variation within the MTBC and additional lineages of clonal pathogens.

A useful tool for investigating the evolution of gene content within a bacterial species is the pangenome (Field et al. 2006; Lapierre and Gogarten 2009; Gordienko et al. 2013; Alcaraz 2014). The pangenome of a bacterial lineage is the collection of all its genes (Tettelin et al. 2005; Gordienko et al. 2013; Lobkovsky et al. 2013; Alcaraz 2014). Within the pangenome, groups of orthologous genes are combined into clusters termed pangenes. It is possible to examine the dynamics of gene loss and gain within a bacterial species by considering the shape of the frequency distribution of pangenes within its pangenome. Each pangene appears in a certain number of strains belonging to the lineage. Some pangenes, termed “rare pangenes,” appear in only one or a small minority of strains; others, termed “core pangenes,” appear in all or the majority of strains belonging to the lineage. Studies of the pangenomes of many bacterial lineages have demonstrated that the frequency distributions of pangenes tend to be bimodal, asymmetrically U-shaped (Hogg et al. 2007; Lefebure and Stanhope 2007; Lapierre and Gogarten 2009; Gordienko et al. 2013). This means that there tend to be many core pangenes and many rare pangenes, but not many pangenes that appear at intermediate frequencies. Core pangenes found in most but not all strains of a species have likely mostly experienced gene loss in the strains from which they are absent. After all, to explain such pangenes through gene gain would require that an introduced gene arise to high frequencies within the species. Given the U-shape of most pangenome distributions, this would imply that many horizontally introduced pangenes rise to high frequencies, but almost never to intermediate frequencies. This seems much less reasonable. At the same time, it is likely that most rare pangenes have been introduced into the strains in which they are found through HGT. After all, in order for a pangene to appear in only one or a few genomes within a species, it is sufficient for one gene gain event to occur. The alternative explanation of gene loss would require multiple events, unless the loss occurred early in the diversification process. Early gene loss is not likely to explain the U-shape of most pangenome distributions, as this would require that a single outlier strain be present within each studied lineage (otherwise one would not expect to find many pangenes present in only a single strain, but nearly no pangenes appearing at intermediate frequencies).

We analyzed extensive whole-genome sequence data from a large collection of clonal and nonclonal pathogenic bacterial species to compare the factors contributing to genetic variation between them. We show that, surprisingly, levels of variation in gene content are similar between the clonal species, which are much less diverged in their gene sequences and undergo little if any recombination and HGT, and some nonclonal species that are much more diverged in their gene sequences and undergo extensive HGT. We further show that this surprisingly extensive variation in gene content within clonal species is driven by gene loss. Our results show that gene loss contributes disproportionally to the generation of genetic variation within clonal bacterial species. Therefore, gene loss needs to be taken into account as a potential dominant contributor to phenotypic variation within these species.

## Materials and Methods

### Data Sets

The protein and genomic sequences used in this study were downloaded from the National Center for Biotechnology Information (NCBI) database (Tatusova et al. 2014). For this study, we focused on bacterial species for which at least ten fully sequenced and annotated genomes were available. Such data were available for four pathogenic species that we found were previously reported to be clonal: The MTBC (Hershberg et al. 2008; Comas and Gagneux 2011; Namouchi et al. 2012), *Yersinia pestis* (Achtman et al. 1999; Chain et al. 2004), *Corynebacterium pseudotuberculosis* (Connor et al. 2007; Soares et al. 2013), and *Francisella tularensis* (Johansson et al. 2004; Nubel et al. 2006; Vogler et al. 2009). In addition, we randomly selected ten pathogenic bacterial species that were not reported to be clonal. Supplementary table S1, Supplementary Material online, lists all bacterial strains used in our research.

After downloading genetic data of the investigated strains, we used information available on the NCBI to manually verify that these strains were indeed fully sequenced and closed. Strains whose genome sequencing process description did not include finishing steps, such as gap closure, were removed from further analysis to prevent computational bias during gene loss analysis due to incomplete sequencing. In addition, we removed strains that are the result of specific manipulations of other strains in the laboratory and genomes that represent multiple clones taken from a single site rather than different strains. The removed strains are listed in supplementary table S2, Supplementary Material online.

We combined *Escherichia coli* and *Shigella* spp. data sets into one group, as studies indicate that *Shigella* is a subspecies of *E. coli* (Rolland et al. 1998; Pupo et al. 2000). From the 13 *Clostridium botulinum* strains available on the NCBI database, we analyzed only ten strains that belong to lineage 1 (supplementary table S1, Supplementary Material online). This was done because the three remaining strains showed remarkably low amino acid identity (AAI) values when compared with other strains from the same species and even when compared with each other (56–60% AAI). Such low AAI values raise issue with the validity of the claim that these strains belong to the same species as the remaining ten strains.

### Classification of the Investigated Bacterial Species into Clonal and Nonclonal

The classification of bacterial species into clonal and nonclonal (or recombining) was initially based on the literature suggesting that the four species we consider clonal are indeed clonal. To further examine this, we wanted to examine whether for each species we see some indication of recombination occurring within their genes. Methods used for detection of the recombinant genes are expected to be sensitive to the length of the investigated sequences. They could also be affected by inclusion of paralogs in the analysis and by differences in the number of sequences used. Therefore, for each bacterial species we analyzed only “core,” single-copy genes (as identified by the pangenomes of these species, see below). We extracted sequences of the core genes from.fna files (files that include annotated nucleotide sequences of all genes in the given organism) of the investigated strains and aligned them using MUSCLE (Edgar 2004) with default parameters. The 900 central positions of each alignment were chosen for the recombination analysis to prevent computational biases due to the differences in gene length. Genes shorter than 900 bp and genes whose alignment had more than 10% gaps within the 900-bp frame were discarded.

To test whether an alignment of orthologous core genes contained statistically significant evidence of recombination, we applied the Pairwise Homoplasy Index (PHI) test (Bruen et al. 2006) using the Phipack software package (Bruen 2005). The following parameters were used: Window size of 100 bp, *P* value computed from 1,000 permutations. We classified an alignment as “recombinant” when the *P* value of the permutation test was lower than 0.05 for PHI test and “nonrecombinant” when it was higher than 0.05. Alignments lacking enough informative sites to perform the recombination tests were considered “noninformative.”

It is important to note that we were not attempting to use PHI to examine rates of recombination. Rather, our sole propose was to examine whether some signal of recombination can be found within the sequences of the different genes. A species for which such a signal was very rarely found is likely to be evolving clonally. At the same time, a species for which many genes show a signal of undergoing homologous recombination is likely not evolving entirely clonally.

### Determining the Relationship between AAI and Genomic Fluidity for the Investigated Bacterial Species

For each bacterial species/group, we used FASTA (Pearson and Lipman 1988) to perform pairwise comparisons of all the strains found within the given group. Orthologous sequences were identified by requiring reciprocal best hits. Following the thresholds set by POGO-DB, for a putatively orthologous pair to be included in amino acid identity calculations it had to have an identity threshold of at least 30% identity across at least 70% of the protein length (Lan et al. 2013). The average amino acid identity (AAI) of a pair of genomes was then calculated as the average percent of AAI across all orthologous protein pairs.

The threshold for the calculation of genomic fluidity was also inspired by the thresholds set by POGO-DB (Lan et al. 2013). In our calculations, however, we used a normalized identity (NI) percent to determine a given protein’s presence or absence for each genome. NI is calculated as follows:

$$\text{NI}=I*\frac{\text{AL}}{\text{QL}},$$

where AL is the alignment length, QL is the query length, and *I* is the percent identity across the aligned region.

For a protein present in one genome to be deemed present in the other genome, it had to find a match in the second genome with at least 50% NI. Genomic fluidity for a pair of genomes *r* and *i* was then calculated as follows:

$$\text{Fluidity}(i,r)=\frac{U_{i}+U_{r}}{T_{i}+T_{r}}$$

where *U_i_* and *U_r_* are the number of genes found to be unique to the query genome *i* and to the reference genome *r*, respectively, and *T_i_* and *T_r_* are the total number of genes in the query genome *i* and the reference genome *r*, respectively.

### Paralog Removal

There are no clear rules as to how paralogs should be treated when generating a pangenome; therefore, their inclusion in the pangenome may generate computational biases. We thus removed all genes that had a paralog within some or all of the genomes analyzed from consideration, prior to generating the pangenomes. To remove paralogs, the protein sequences from each genome were compared against all other proteins within that genome, using FASTA (Pearson and Lipman 1988). Query sequences that identify within the genome sequences other than themselves were dubbed “paralog sequences” and removed from the file, together with the sequences they recognize. As a threshold for considering sequences as matching we required at least 80% NI between the sequences.

After identifying paralogs we used FASTA (Pearson and Lipman 1988) to remove all paralog-related sequences from the files as well. Paralog-related sequences are coding gene sequences that have no paralogs in a given genome but identify with the paralog sequences in another genome above the threshold of 80% NI. Because of paralog sequence removal those sequences will be underrepresented in the pangenome and their inclusion in the calculations will generate artifacts. To maximize the sensitivity of the paralog and paralog-related sequences removal step, we performed sequence alignment and comparison at both the protein and nucleotide levels. Only sequences that were found to be nonparalog and nonparalog-related at both the protein and DNA levels were used for pangenome construction.

### Pangenome Construction

After removal of all paralog and paralog-related genes, we constructed a library that contains the annotated protein sequence files of each of the considered strains. One of the protein files was chosen randomly as the initial database file and a second file was compared with the initial database using FASTA (Pearson and Lipman 1988). A query protein sequence that identified a protein sequence within the database was considered to be present in both organisms. A query that did not match any sequence in the database was considered to be absent from the database and was added to the initial database. As a threshold for considering sequences as matching we required 80% NI. After all proteins within the query genome were compared with the database, a third file was compared with the expanded database and the process was repeated over all protein sequence files within the library.

A shorter protein query compared with a longer database protein will be more likely to pass the 80% NI threshold than a longer query protein compared with a short database protein. This may insert biases into the pangenome construction, by which the generated pangenome will change depending on the order in which files were compared with each other. To prevent such artifacts, the pangenome that was generated as described above was compared with itself using FASTA (Pearson and Lipman 1988). Pangenes that matched pangenes other than themselves above the threshold described above were combined into one pangene.

**Fig. 2.—:**
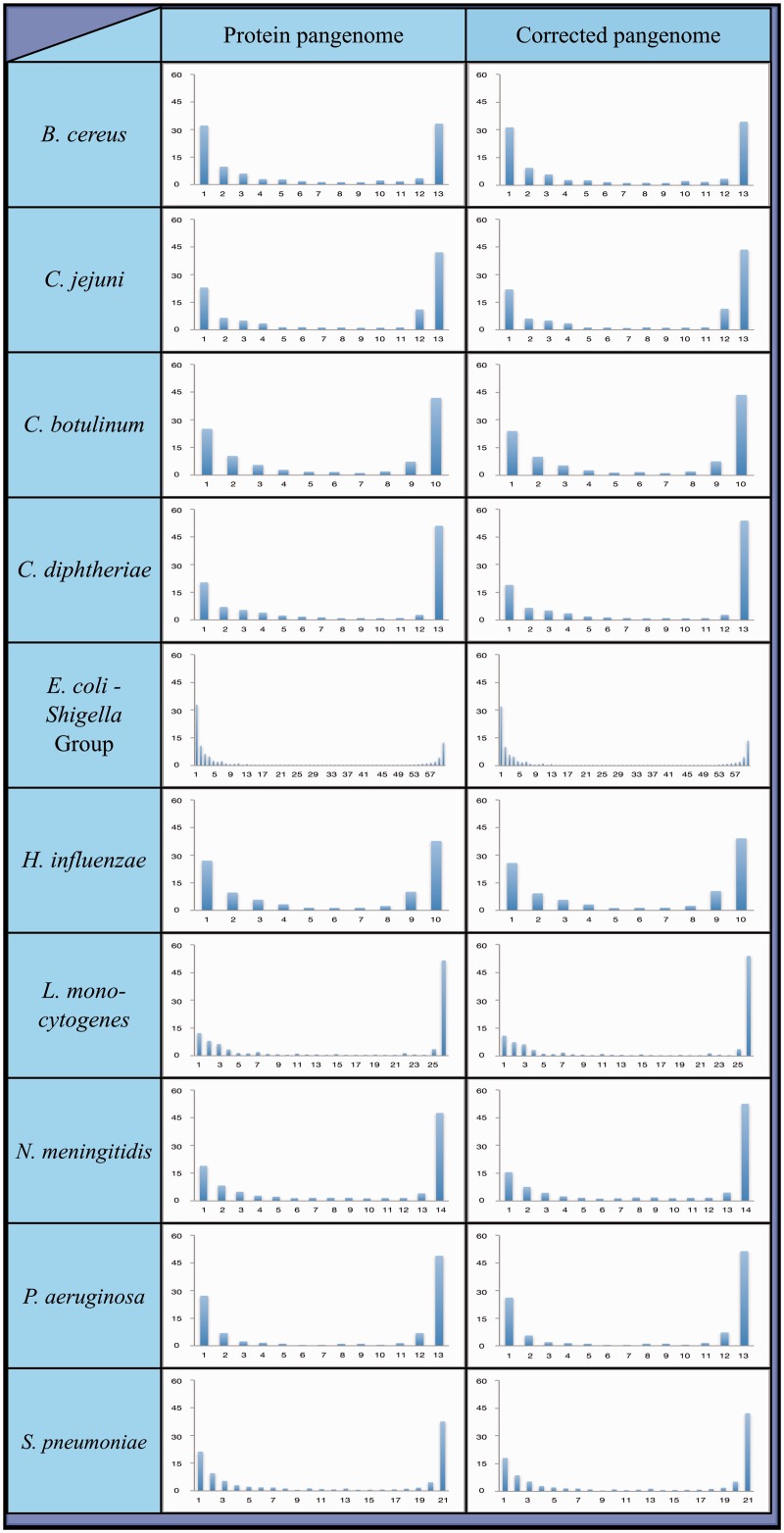
Pangenome plots of ten recombining bacterial species. To generate these plots, all strains within a species were compared and orthologous genes were clustered together into groups. This allows for the calculation of the frequency with which each cluster of orthologous genes (referred to as a “pangene”) is found among members of its species. Depicted are the distributions of frequencies with which pangenes are found within each species. Two plots are provided for each species: Left: Protein pangenome plot—generated based on annotated protein sequences; Right: HGT-artifact corrected pangenome plot—the protein pangenome was compared with the full DNA sequences of the strains from which it was generated. Pangenes appearing in less than 50% of strains within a species at the annotated protein level, but in more than 75% of the strains at the whole DNA level, were removed from the plot.

**Fig. 3.—:**
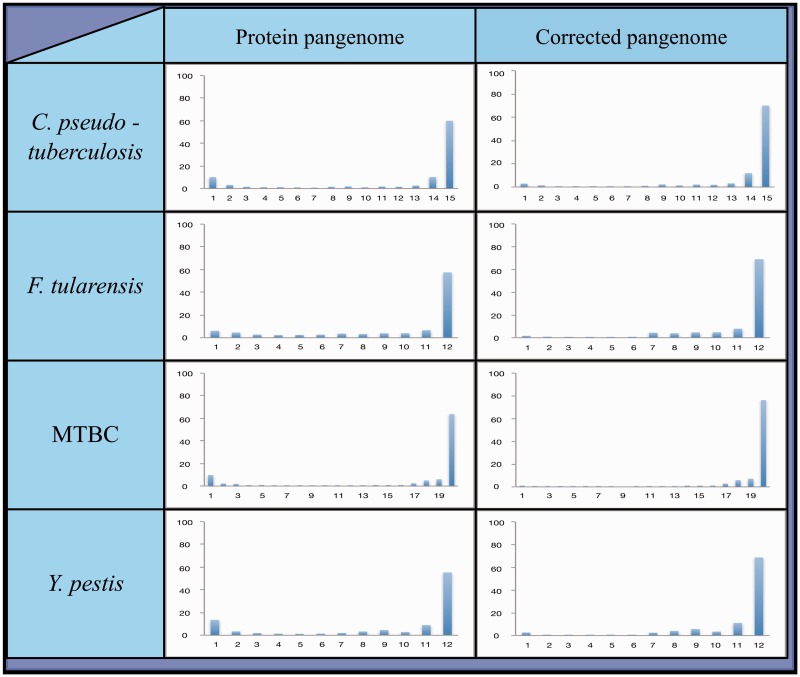
Pangenome plots of four clonal bacterial species. To generate these plots, all strains within a species were compared and orthologous genes were clustered together into groups. This allows for the calculation of the frequency with which each cluster of orthologous genes (referred to as a “pangene”) is found among members of its species. Depicted are the distributions of frequencies with which pangenes are found within each species. Two plots are provided for each species: Left: Protein pangenome plot—generated based on annotated protein sequences; Right: HGT-artifact corrected pangenome plot—the protein pangenome was compared with the full DNA sequences of the strains from which it was generated. Pangenes appearing in less than 50% of strains within a species at the annotated protein level, but in more than 75% of the strains at the whole DNA level, were removed from the plot.

The results of the FASTA alignments were used to calculate the number of strains in which each pangene was found within a species. The distribution of these numbers was used to generate the species’ pangenome plot (see figs. 2 and 3, left column).

### Gene-Gain (HGT) Analysis

This analysis was carried out to determine whether “rare” pangenes (found in 25% or less genomes) were likely to be the results of gene gain through HGT. To this end, the initial pangenome was compared with the whole DNA sequence files of the considered bacterial strains using the FASTA program TFASTX (which allows for the comparison of a protein query against a DNA sequence) (Pearson and Lipman 1988). Pangenes that are “rare” due to their being introduced into the species through HGT will be absent from the genomes in which they are not found at the DNA level as well. If, however, a pangene was identified as “rare” based on protein annotations but is found within many additional genomes when compared against whole DNA, it is not likely to be the result of HGT. Rather, such pangenes are very likely to represent misannotations occurring only in one or a few genomes. If according to the whole-genome level TFASTX alignments a pangene was found in over 75% of the strains of a species, but according to the protein-annotation level FASTA alignments it was found in less than 50% of the strains, it was dubbed as an artifact and removed from the initial pangenome. The pangenomes after removal of the possible artifacts are shown in the plots presented in figures 2 and 3, right column.

### Estimating the Proportion of Lost “Near Core” Pangenes that Are Maintained as Pseudogenes

For the “near core” pangenes, we asked what proportion of genomes from which these genes were absent at the protein level still contained them at the whole genome DNA level. To this end, the protein sequences of these pangenes were compared with the DNA sequences of the genomes from which they were found to be absent at the annotated protein level using TFASTX.

To determine whether a pangene absent at the annotated protein level was nevertheless present at the DNA level, we demanded a threshold of 80% identity across the entire sequence length of the query protein. Pseudogene Conservation percent (PCP) of “core” genes was then calculated as follows:

$$\text{PCP}=\frac{(\sum_{\text{i}=1}^{\text{n}}P_{i})}{\left(\sum_{\text{i}=1}^{\text{n}}A_{i}\right)},$$

where *n* is the number of “near core” pangenes, *P_i_* is the number of genomes in which pangene *i* is found at the DNA (but not the protein) level, and *A_i_* is the total number of genomes in which pangene *i* is not found at the annotated protein level.

### Generation of RAST-Annotation-Based Pangenomes

To obtain consistent annotations of the investigated bacterial pangenomes, we used the RAST annotation webserver (Aziz et al. 2008; Overbeek et al. 2014), with default parameters. We then constructed the protein-sequence-based pangenomes, based on these automatically generated annotations, and corrected them for the presence of possible HGT artifacts (as described for the pangenomes generated based on NCBI annotations, see supplementary figs. S1 and S2, Supplementary Material online, left columns).

## Results

### Clonal and Nonclonal Bacterial Species

We extracted from the NCBI (Tatusova et al. 2014) the full genome sequences and protein-coding gene annotations of four pathogenic bacterial species that were previously designated as clonal: The MTBC (Hershberg et al. 2008; Comas and Gagneux 2011; Namouchi et al. 2012), *Yersinia pestis* (Achtman et al. 1999; Chain et al. 2004), *C. pseudotuberculosis* (Connor et al. 2007; Soares et al. 2013), and *F. tularensis* (Johansson et al. 2004; Nubel et al. 2006; Vogler et al. 2009)*.* For each of these species, there were at least ten fully sequenced strains available in the database. Additionally, we extracted from the NCBI the full genome sequences and protein-coding gene annotations of ten additional pathogenic species that have not been described as being clonal. These species were selected at random from those species that have at least ten fully sequenced strains and we made sure not to include any two species sharing the same genera. A summary of all bacterial species analyzed and of the number of genomes analyzed in each species is given in table 1.

**Table 1:** Levels of Variation in the Sequences of Genes and in Gene Content within the 14 Examined Species and Correlations between These Types of Variation

Species Name	No. of Analyzed Genomes	Minimal AAI	Maximal Fluidity	Spearman’s *ρ*^a^	% of Increase in Genomic Fluidity per 1% Decrease in AAI	% of Increase in Genomic Fluidity per 1% Decrease in AAI for Strains with AAI >99.5%
*Bacillus cereus*	13	93.05	0.225	−0.843 (*P *≪ 0.0001)	1.51	N/A
*Campylobacter jejuni*	13	95.72	0.279	−0.812 (*P *≪ 0.0001)	4.89	7.32
*Clostridium botulinum*	10	95.60	0.191	−0.638 (*P *≪ 0.0001)	2.65	N/A
*Corynebacterium diphtheriae*	13	98.00	0.147	−0.474 (*P *≪ 0.0001)	4.95	N/A
*Corynebacterium pseudotuberculosis*^b^	15	98.59	0.103	−0.746 (*P *≪ 0.0001)	4.03	15.46
*Eschirechia coli–Shigella*	60	97.37	0.287	−0.677 (*P *≪ 0.0001)	5.66	13.65
*Francisella tularensis*^b^	12	98.84	0.168	−0.748 (*P *≪ 0.0001)	6.33	8.95
*Haemophilus influenzae*	10	95.98	0.216	−0.417 (*P* = 0.0056)	2.34	N/A
*Listeria monocytogenes*	26	96.06	0.133	−0.825 (*P *≪ 0.0001)	1.71	10.14
MTBC^b^	20	99.62	0.140	−0.820 (*P *≪ 0.0001)	26.16	26.16
*Neisseria meningitides*	14	97.52	0.162	−0.785 (*P *≪ 0.0001)	5.67	N/A
*Pseudomonas aeruginosa*	13	94.57	0.169	−0.759 (*P *≪ 0.0001)	1.77	N/A
*Streptococcus pneumonia*	21	97.67	0.187	−0.567 (*P *≪ 0.0001)	5.56	N/A
*Yersinia pestis*^b^	12	99.65	0.164	−0.632 (*P *≪ 0.0001)	31.31	31.31

Note.—N/A denotes species that have few or no pairs of strains with AAI >99.5%.

^a^Spearman’s ρ coefficient calculated for correlation between AAI and genomic fluidity for considered bacterial species. P-value is shown in parentheses.

^b^Clonal species.

To examine whether the species we designated as nonclonal indeed seem to undergo homologous recombination we used the Phipack software package (Bruen 2005), which we applied to all single-copy genes conserved across all members of a species (Materials and Methods). This allowed us to determine whether for each of the studied species there is any signal of recombination occurring. It is important to note that we cannot use this program to compare relative rates of recombination between the different nonclonal species. However, if we see that a substantial number of genes within a species show a signal of having undergone recombination, we can be more confident that the species in question is not evolving clonally.

For two of the species that were previously designated as clonal (*M. tuberculosis* and *Y. pestis*), levels of sequence variation were extremely low (see below). This left us with very little power to detect recombination within these species. For the two remaining species that were previously designated as clonal (*F. tularensis* and *C. pseudotuberculosis*), we were able to test a substantial number of genes for recombination; almost no genes showed any signal of having recombined during the evolution of the species (supplementary table S3, Supplementary Material online)*.* This supports their prior designation as clonal. In contrast, for the ten species that were not previously designated as clonal we find substantial numbers of genes that show significant signals of recombination (supplementary table S3, Supplementary Material online). This suggests that these species are indeed not evolving clonally and that they each experience some recombination. We will therefore from now on refer to the four species that were previously designated as evolving clonally as “clonal” and to the remaining ten species as “nonclonal.”

### Sharper Changes in Gene Content Are Observed during Early Stages of Diversification

Within each species we compared the sequences of all protein-coding genes in a pairwise manner to estimate innerspecies diversity. For each orthologous gene pair, we calculated its amino acid percent identity. Based on these comparisons of orthologous protein pairs, we calculated for each pair of genomes the average amino acid identity (AAI) (Konstantinidis and Tiedje 2005; Lan et al. 2013) across all their orthologous proteins. As this metric takes into account information from many loci, it provides a good estimator of the level of gene-sequence diversity between genome pairs. We then calculated the minimal AAI within each bacterial species, which is akin to calculating the maximal protein-coding gene sequence diversity between members of a species. We found variation in minimal AAI between species ranging from 93.05% in *Bacillus cereus* to 99.65% in *Yersinia pestis* (table 1). Clonal species were characterized with high minimal AAI values (ranging between 98.59% and 99.65%, table 1). Nonclonal species had a much broader range of minimal AAIs (ranging between 93.05% and 98%) and none of them had a minimal AAI that was as high as that of any of the examined clonal species.

Next, we estimated levels of gene-content diversity between the genome pairs using the genomic fluidity metric (see Materials and Methods; Kislyuk et al. 2011; Lan et al. 2013). For a pair of genomes, genomic fluidity denotes the fraction of genes that are unique to only one of the genomes out of the total of genes present in the two genomes (Lan et al. 2013). Therefore, lower genomic fluidity corresponds to a higher similarity in gene content. Maximal genomic fluidity was also found to vary greatly between the investigated species, ranging from 0.103 in *C. pseudotuberculosis* to 0.287 in the *E. coli–Shigella* group (table 1). In contrast to what we saw for minimal AAI, maximal genomic fluidity was not always lowest for the clonal species. This indicates that although clonal species tend to be the least diverged in their gene sequences, among all species studied, they are not always the least diverged in their gene content.

Within each species, we examined the relationship between levels of variation in the sequence of orthologous genes (as measured by AAI) and genomic fluidity. We found that across all investigated bacterial species there are highly significant negative correlations between AAI and genomic fluidity (table 1, *P *≪ 0.001, for all comparisons, excluding *Haemophilus influenza* for which *P* = 0.0056, according to a Spearman correlation test). This suggests that, within all bacterial species, levels of variation in gene content increase in a clocklike manner alongside changes in the sequences of orthologous genes. At the same time, the degree of change in gene content per AAI unit varies greatly between different species (table 1). The most extreme levels of change in genomic fluidity per AAI unit are observed in the two clonal species with the lowest levels of sequence divergence: *Y. pestis* and the MTBC. Based on linear regression of genomic fluidity against AAI, within *Y. pestis* a change of 1% in AAI corresponds to an approximately 31% change in genomic fluidity; within the MTBC a similar 1% change in AAI corresponds to an approximately 26% change in genomic fluidity*.* For comparison sake, a 1% change in AAI corresponds to only an approximately 5.7% change in genomic fluidity in the *E. coli–Shigella* group and an approximately 1.5% change in genomic fluidity in *B. cereus* (table 1)*.*

Some of the nonclonal species show a very wide range of AAI, meaning that some genome pairs within the species are highly similar whereas others are more diverged. Within such species, when we examine the relationship between AAI and genome fluidity for genome pairs that are very similar (AAI > 99.5%), we find a relationship between AAI and fluidity more similar to that seen in the least diverged clonal species than to that seen in the more diverged genome pairs of these species (table 1). This means we see sharper changes in gene content between closely related genome pairs, irrespective of the species to which they belong and to whether that species is clonal or not. These findings imply that rates of change in gene content are not constant for given species and may change over time. At the beginning of the diversification process, bacteria seem to experience sharper changes in gene content; within the nonclonal species, this effect levels out during later stages of diversification.

### Clonal Pathogens Can Be Highly Diverged in Their Gene Content, Given Their Low Levels of Gene Sequence Variation and Lack of Recombination

As mentioned above, within species AAI correlates very well with gene fluidity (*P *≪ 0.001, for all comparisons, excluding *H. influenza* for which *P* = 0.0056; table 1). This implies that gene content changes are carried out in a clocklike manner alongside changes in the sequences of genes. At the same time, when we carried out our comparisons across species, we found only a marginally significant correlation between the minimal AAI of a species and its maximal gene fluidity (*P* = 0.034, according to the Spearman Rank correlation test; table 1). In other words, the expected trend by which species that are more diverged at the level of gene sequences are also more diverged in their gene content seems to be weaker than we might expect.

Of particular interest to us, we observed that the four least diverged, clonal bacterial species do not have much lower levels of variation in gene content than many of the more diverged nonclonal species (table 1). Indeed, in some cases levels of gene content variation within clonal species can be higher than those of more diverged nonclonal species. For example, *Y. pestis,* that has a minimal AAI of 99.65%, has a maximal genomic fluidity of 16.4%. This is higher than the maximal genomic fluidity of *Listeria monocytogenes, Neisseria meningitidis* and *Corynebacterium diphtheria*, all of which are substantially more diverged in the sequences of their genes (table 1).

### Generation of Species Pangenomes Based on Annotated Protein-Coding Gene Sequences

The results presented so far suggest that clonal pathogens, which seem to be extremely nondiverged in their gene sequences, are nevertheless quite diverged in their gene content. As clonal bacterial species are thought to undergo little if any HGT, such divergence in gene content likely arises mostly through gene loss.

To examine the contribution of gene loss and HGT to the changes in gene content in the examined bacterial species, we generated and analyzed the pangenomes of the 14 bacterial species. As described in the Introduction, pangene frequency distributions (also referred to as “pangenome plots”) tend to follow a bimodal distribution (Hogg et al. 2007; Lefebure and Stanhope 2007; Lapierre and Gogarten 2009; Gordienko et al. 2013), with many rare pangenes and many common pangenes, but very little pangenes of intermediate frequencies. Those pangenes that appear in only a small fraction of strains within a species were likely introduced into the species through HGT. At the same time, pangenes appearing in most, but not all, strains of a species are likely part of the “core” genome of the species that were lost in some strains. It is therefore possible to examine the shape of the pangenome plot in order to investigate the relative contributions of gene loss and HGT to changes in gene content.

To generate a pangenome for a given species, we chose an annotated protein sequence file from one strain at random as the initial pangenome database. We then started by comparing an annotated protein sequence file from a second randomly selected strain to this initial database file, using FASTA (Pearson and Lipman 1988). A query that identified a protein sequence within the initial database was considered as a possible ortholog and added to this pangene group. A query that did not identify any protein within the database was considered to be absent from the database and its sequence was added to the database file. After all the proteins within the second file were compared with the database, a third sequence file was compared with the expanded database in the same fashion. The process was repeated until all protein sequence files had been compared. The resulting initial pangenome was then compared with itself to prevent a computational bias that could arise from choosing a random genome to serve as an initial database for the pangenome construction (see Materials and Methods). The resulting pangenomes are shown in figures 2 and 3, left columns.

### Very Little HGT within Clonal Species

The pangenes within each pangenome were divided into three groups: “Rare”—appearing in 25% or less of the genomes that constitute the pangenome; “core”—appearing in all the genomes within the pangenome; and “near core”—appearing in more than 75% of genomes, but not in all genomes.

Clonal bacterial species are expected to undergo little if any HGT. Therefore, we expected to find only a small number of pangenes in the “rare” group, within clonal species. As expected, we found a substantially smaller proportion of “rare” pangenes for the clonal species (fig. 3 left panel, compared with fig. 2 left panel and supplementary table S4, Supplementary Material online).

In our analysis, we assume that “rare” pangenes represent instances of HGT. However, the pangenomes we generated depend on the protein annotation of genomes. It is therefore possible that some “rare” pangenes could be the result of misannotation of a nonfunctional gene sequence as a functional gene, in only one or a few strains. To remove such false “rare” pangenes from consideration, we compared the pangenes of each bacterial lineage with full DNA sequences of the strains that were used to generate the pangenome. If an annotated protein sequence found in only one or a few organisms were truly a result of HGT, we would expect to find it in a few organisms at the DNA level as well. However, if the DNA sequence of the gene is present in many additional genomes from the same species, then this sequence was clearly not truly introduced into that species horizontally.

After comparisons to the full genomic sequences we redrew the pangenome plots using the following rule: If a given pangene was found in 50% of the organisms or less at the annotated protein level, while at the genomic DNA level it was found in 75% of the genomes or more, then it was removed from the pangenome plot (figs. 2 and 3, right columns). Following this correction we could examine the frequency of “rare” pangenes within each species to estimate the degree to which HGT contributes to gene content variation within each species. As expected, we found much higher frequencies of “rare” panges in the nonclonal (∼30–∼66%), compared with the clonal species (∼2–∼6%; table 2). This clearly demonstrates that there is very little HGT in the clonal species.

**Table 2:** Summary of Pangene Frequencies within the Corrected Pangenomes^a^ of the 14 Studied Species

Species	Pangenome Size^b^	“Rare” Pangenes^c^	“Near Core”Pangenes^d^	“Core” Pangenes^e^
*Bacillus cereus*	9,769	46.67	5.39	34.34
*Campylobacter jejuni*	2,465	33.02	12.78	43.53
*Clostridium botulinum*	5,417	39.47	9.71	43.53
*Corynebacterium diphtheria*	3,122	30.88	3.97	53.78
*Corynebacterium pseudotuberculosis*^f^	2,209	5.57	16.79	70.03
*Eschirechia coli-Shigella*	11,305	65.61	14.14	13.51
*Francisella tularensis*^f^	1,484	3.23	12.74	69.00
*Haemophilus influenzae*	2,681	40.69	12.94	39.09
*Listeria monocytogenes*	4,197	32.14	6.70	53.80
MTBC^f^	3,752	1.81	17.11	76.28
*Neisseria meningitidis*	2,463	30.00	7.96	52.46
*Pseudomonas aeruginosa*	8,585	33.72	8.77	51.37
*Streptococcus pneumoniae*	2,960	37.13	9.53	42.23
*Yersinia pestis*^f^	3,690	3.69	14.63	68.64

^a^The corrected pangenome is constructed by generating the pangenome based on annotated protein-coding genes and then removing pangenes if they are found in less than 50% of strains at the protein level but in more than 75% of strains at the whole DNA level.

^b^Number of pangenes (orthologous gene clusters) within pangenome.

^c^% of pangenes that are found in less than 25% of strains of a species.

^d^% of pangenes that are found in over 75% of strains of a species, but are not found in all strains.

^e^% of pangenes found in all strains of a species.

^f^Clonal species.

### Relatively High Abundance of Near Core Pangenes within the Clonal Species, Indicating Relatively Prevalent Gene Loss

Pangenes appearing in nearly all investigated genomes are likely to have undergone gene loss in the genomes from which they are absent. To investigate gene loss, we therefore focused on the “near core” pangenes. This group constitutes approximately 13% to approximately 17% of pangenes within the pangenomes of the four clonal species (table 2). This percentage is higher than the percent of the “near core” pangenes found within the pangenomes of seven of the ten nonclonal species examined (table 2). The fact that extremely nondiverged clonal species have a higher fraction of “near core” pangenes than is found within so many of the nonclonal species serves to highlight the extremely high relative contribution of gene loss to the evolution of these clonal pathogens.

Cases in which a “near core” pangene is absent from a certain strain’s genome most likely represent instances of gene loss within that strain. We counted how many such instances occurred for each strain within the 14 studied bacterial species. We then divided this number by the total number of genes within each strain that were used to construct the species pangenome. This allowed us to examine how many genes are clearly lost within each strain, normalized by that strain’s gene count. For convenience, we will refer to this number as the percentage of genes lost.

Within each species there was great variation between strains in the percentage of genes lost (see supplementary table S1, Supplementary Material online). In many instances this likely represents the fact that some strains are more diverged from the bulk of the other strains, allowing them more time to lose core genes. To examine this, we calculated for each strain within each species its pairwise AAI compared with each of the other strains of that species. We then averaged these AAI values across all pairwise comparisons, to obtain the average AAI (aAAI) of that strain against all other strains. The less similar a strain is to more of the other strains within its species, the lower its aAAI will be. We found significant negative correlations between aAAI and the percentage of genes lost for 7 of the 14 examined species, including all four of the clonal species (*P* < 0.05 according to a Spearman correlation test; see supplementary table S5, Supplementary Material online). In the seven remaining species however, no significant correlation was found between sequence divergence, as measured by aAAI, and the percent of genes lost (see supplementary table S5, Supplementary Material online). This could reflect changes in rates of gene loss occurring within these species. For example, we observed no significant correlation between aAAI and the percentage of gene loss within the *E. coli*–*Shigella* lineage. This lack of correlation may be explained at least in part by our previous results showing that *Shigella* experiences accelerated gene loss relative to other *E. coli* strains (Hershberg et al. 2007). Supporting this, the percentage of genes lost ranges between 8.9% and 23.3% for all *Shigella* strains, except one. At the same time, for *E. coli* strains the highest percentage of genes lost is 6.2%.

The maximal percentage of genes lost within each species tends to be relatively high for the clonal species. For example, the MTBC has a higher maximal percentage of genes lost than eight of the ten nonclonal species (nine, if one excludes *Shigella* strains within the *E. coli*/*Shigella* lineage). At the same time, *Y. pestis* and *F. tularensis* have a higher percentage of genes lost than six of the ten nonclonal species. Even for *C. pseudotuberculosis*, which seems to have lost the least genes among clonal pathogens, the maximal percentage of genes lost is still higher than that of three of the ten nonclonal species (table 3). The fact that the clonal pathogens have a higher percentage of genes lost than many of the nonclonal ones stands in sharp contrast to the fact that clonal species have the lowest diversity in gene sequences (as measured by minimal AAI).

**Table 3:** Maximal Percentage of Lost “Near Core” Genes

Species	Maximal Gene Loss (%)^a^ NCBI Annotations^b^	Maximal Gene Loss (%)^a^ RAST Annotations^c^
*Bacillus cereus*	3.3	2.0
*Campylobacter jejuni*	10.2	5.8
*Clostridium botulinum*	5.5	3.3
*Corynebacterium diphtheriae*	1.7	1.6
*Corynebacterium pseudotuberculosis*^d^	4.1	3.7
*Eschirechia coli–Shigella*	*Eschirechia coli*: 6.2 *Shigella* spp.: 23.2	*Eschirechia coli*: 2.3 *Shigella* spp.: 17.3
*Francisella tularensis*^d^	6.1	4.9
*Haemophilus influenzae*	9.2	4.4
*Listeria monocytogenes*	3.8	3.3
MTBC^d^	9.8	3.2
*Neisseria meningitidis*	2.7	3.6
*Pseudomonas aeruginosa*	7.0	8.8
*Streptococcus pneumoniae*	5.1	2.7
*Yersinia pestis*^d^	6.9	5.6

^a^For each strain we calculated the percentage of genes that were lost from that genome as 100*L/T, where L is the number of “near core” core pangenes that are absent from the genome and T is the total number of genes present within that genome, that were used to construct the species pangenome (see Materials and Methods). Given in this table are the maximal values obtained for each species.

^b^Pangenomes were generated using the annotation provided by the NCBI alongside each genome sequence

^c^Pangenomes were generated using the annotations obtained by using RAST. Annotations were length corrected (see text).

^d^Clonal species.

Combined these results demonstrate that considering their extremely low levels of gene sequence diversity, clonal species tend to lose a relatively very high number of genes. Given the fact that they are so nondiverged in their gene sequences and undergo little to no HGT, the relative contribution of gene loss to the generation of genetic variation is especially high for such clonal species.

### Frequent Maintenance of Pseudogenes within the Genomes of Clonal Pathogens

A deactivated gene that has lost its function may be either removed completely or maintained as a psuedogene. To estimate the frequency with which genes that are lost are maintained as pseudogenes within each species, we focused on the “near core” pangenes (found at the annotated protein level in more than 75% of genomes, but not in all genomes of the species). As we discussed above, such pangenes were likely lost from the strains from which they are absent. We compared the sequences of these pangenes with the full genome sequences of the strains from which they were found to be absent. We then asked in what proportion of genomes from which such a pangene is absent at the protein-level, it is still found at the DNA level (see Materials and Methods). We found that in the clonal species, a core pangene that is absent from a genome at the protein level is found within that genome at the DNA level in 68–97% of cases (table 4). Only three of the ten nonclonal species had a frequency of pseudogene maintenance that fell within this high range (ranging between 71.5% and 75.3% for *S. pneumoniae, C. jejuni*, and *H. influenza*; table 4). *Neisseria meningitidis* also seems to maintain a relatively large fraction of deactivated genes (∼61%). For the remaining six nonclonal species, the frequency of maintenance of pseudogenes ranges between 32% and 52% of “near core” pangenes (table 4). Combined, these results suggest that clonal pathogens tend to maintain very high numbers of the genes they lose as pseudogenes. At the same time, the frequency of maintenance of pseudogenes in the nonclonal species varies greatly and is usually lower than that observed in the clonal pathogens.

**Table 4:** Frequencies of Pseudogene Maintenance

Species	PCP (%)^a^
*Bacillus cereus*	40.17
*Campylobacter jejuni*	71.84
*Clostridium botulinum*	51.79
*Corynebacterium diphtheriae*	47.47
*Corynebacterium pseudotuberculosis*^b^	96.74
*Eschirechia coli–Shigella*	44.67
*Francisella tularensis*^b^	95.00
*Haemophilus influenzae*	75.30
*Listeria monocytogenes*	39.07
MTBC^b^	84.56
*Neisseria meningitidis*	60.99
*Pseudomonas aeruginosa*	37.67
*Streptococcus pneumoniae*	71.49
*Yersinia pestis*^b^	67.56

^a^The PCP metric was calculated by focusing on pangenes found, at the protein level, in 75% or more of strains within each species, but not in all strains of that species. PCP was calculated as the proportion of genomes from which such a pangene was absent at the protein level, but was still found at the DNA level.

^b^Clonal species.

### Observed Trends Are Not the Result of Annotation Biases

In our analyses so far, we have relied on the annotation provided with the different genome sequences. Such annotations are often likely better than those that can be obtained using an automated annotation program. This is because the annotations provided alongside the genome sequence are usually also reliant on additional biological knowledge of the sequenced organisms. However, the quality of the annotations provided with the different genome sequences may vary which could potentially lead to biases in our results. To examine this, we also automatically annotated each of the genomes we analyzed using the RAST annotation software (Aziz et al. 2008; Overbeek et al. 2014). We then regenerated our pangenomes using these annotations (Materials and Methods, supplementary figs. S1 and S2, Supplementary Material online, left column).

Automated gene annotation programs may annotate pseudogenes as functional genes if their sequences remain long enough despite of their loss-of-function nonsense mutations. To account for this limitation of the automated annotation programs, we examined the lengths of genes within each pangene cluster. If a gene was found to be shorter than two-thirds of the length of the longest gene sequence within that pangene cluster, we considered this gene to have been lost from its genome. The rational here was that loss of over a third of the length of a gene is quite likely to lead to loss of that gene’s function, or at least to a dramatic change of function. We then redrew each of the species’ pangenomes (see supplementary figs. S1 and S2, Supplementary Material online, right columns).

The pangenomes we find using these “length-corrected” RAST annotations are qualitatively very similar to the pangenomes generated using the annotations provided with the genome sequences. Specifically, we find a much smaller proportion of “rare” pangenes within the clonal species, compared with the nonclonal ones (see supplementary table S6, Supplementary Material online). As before, this indicates that clonal species tend to undergo much less HGT. We also find that the proportion of “near core” pangenes tends to be relatively high for the clonal species. The only nonclonal species that has a proportion of “near core” pangenes that is comparable to those of the clonal species is the *E. coli–Shigella* group (supplementary table S6, Supplementary Material online).

When we examine how many “near core” pangenes are lost from each strain within each species, we also find results that are similar to those observed before (table 3). Namely, the maximal percentage of genes lost per strain is often relatively high for the clonal species.

Combined these results show that the trends we observe remain consistent whether we use the annotations provided with the genome sequences, or whether we consistently reannotate the sequences ourselves. We can therefore confidently say that clonal species tend to undergo little HGT and that they tend to lose a disproportionate amount of genes given their low levels of gene sequence variation.

## Discussion

Our results show that, within the 14 studied species, clonal pathogenic species tend to be less diverged in their gene sequences, compared with nonclonal pathogenic species. Additionally, the pangenomes of the nonclonal species have a high fraction of “rare” pangenes, whereas almost no such “rare” pangenes are found within the pangenomes of the clonal species. This indicates that gene gain through HGT contributes more to gene content variation within the nonclonal species. Yet, despite lower levels of gene sequence variation and HGT-driven gene gain within the clonal species, they have levels of gene content variation comparable to those found in many of the nonclonal species.

Why would strains of clonal species lose so many genes as to make levels of gene content variation within them comparable to those of more gene sequence diverged nonclonal species? One likely contributing factor is a lag between the appearance of certain slightly deleterious mutations and their removal from the population. Such a lag is thought to explain the observation that more closely related bacterial genomes tend to have higher ratios of the rates of nonsynonymous (change the amino acid sequence) and synonymous (do not change the amino acid sequence) protein-coding substitutions (d*N*/d*S*) (Rocha et al. 2006). As nonsynonymous substitutions are more likely to be functional, they will more often be affected by natural selection. A lag in the effects of purifying selection leads to a higher proportion of such nonsynonymous substitutions among closely related genomes (Rocha et al. 2006). Such a lag in the removal of slightly deleterious mutations may also explain why we observed sharper changes in gene content for less diverged strains, irrespective of whether such strains belonged to clonal or nonclonal species. Slightly deleterious gene loss events will take time to be removed by selection, and hence a larger proportion of them will be maintained between closely related genomes. As the clonal species tend to be less diverged in their gene sequences, which may reflect a shorter time of divergence, it is reasonable that such an effect would be more pronounced on the clonal compared with the nonclonal species.

It is important to note however that lack of time to remove deleterious gene deactivation mutations cannot explain why we find that clonal pathogens tend to lose even more genes than many nonclonal species that are much more sequence diverged than they are. It is therefore likely that there are also certain intrinsic traits of clonal species that reduce the efficacy with which natural selection removes deleterious mutations from their populations, in a manner that is not time-dependent. Low recombination rates among clonal species can lead to reductions in effective population size (*N*_e_) (Lynch 2007; Hershberg et al. 2008; Hershberg and Petrov 2010). Additionally, for some of the clonal pathogens (e.g., *M. tuberculosis*) infective dosage is thought to be quite small which may lead to repeat bottlenecking associated with passage from host to host (Smith et al. 2006; Hershberg et al. 2008). This could further contribute to the decrease in *N*_e_. When *N*_e_ is small, the power of stochastic forces such as genetic drift and draft to affect patterns of variation increases relative to the power of natural selection. Thus, when *N*_e_ is small, natural selection becomes relaxed and allows for the accumulation of genetic changes that are deleterious and that otherwise would have been purged from the population (Lynch 2007; Hershberg et al. 2008). This in turn could cause deleterious gene deactivation substitutions that would be removed in an organism with higher *N*_e_ to be maintained in the clonal species for which *N*_e_ is smaller.

It should be noted that the two explanations above are not mutually exclusive. On the contrary, there can be an additive effect. The selection process in general may not be very efficient over short evolutionary time scales, and in the clonal bacteria it is even less effective because of such factors as low recombination rates and small infective dosages. This could explain why clonal species lose so many genes, even though they are so nondiverged in their gene sequences.

Although it may be tempting to attribute much of the gene loss observed in the clonal species to adaptation due to the recent shifts in lifestyle, we do not think this is supported by our data. We observed very strong correlations for all studied species between levels of gene content variation and levels of gene sequence variation. This suggests that gene content variation (that is generated in the clonal species almost exclusively through gene loss) occurs in a clocklike manner along changes in gene sequences. It does not seem likely that adaptation would occur in such a clocklike manner. It is therefore more likely that it is relaxed purifying selection, rather than increased positive selection that drives most gene loss in both the clonal and nonclonal species examined. It is important to note, however, that even if most of the gene loss occurring within the clonal species is nonadaptive, it does not mean that such gene loss events cannot contribute to phenotypic variation. Natural selection will not affect gene loss events that are entirely nonfunctional, as only gene loss events carrying some function will affect fitness. Therefore, for changes in the intensity of selection to affect the proportion of gene loss events that are allowed to persist, many of these gene loss events would have to carry some phenotypic effects. Although the phenotypic effects of gene loss events may often not be initially adaptive for the bacteria carrying them, such gene loss events could still greatly influence the phenotypic variation observed between different strains of the clonal bacterial species.

It is interesting to speculate as to what would happen if the clonal species continue to evolve clonally. Will they undergo severe genome-reduction? This seems quite likely, as the continuous evolution in the absence of recombination should lead to continuous relaxed selection and increased genetic drift and draft. This in turn should lead to a Muller’s ratchet type effect by which genes are continuously lost (McCutcheon and Moran 2012). A glimpse into this future may be provided by *Mycobacterium leprae*, a distant relative of the MTBC. *M. leprae* has been evolving clonally (Monot et al. 2005) and is thought to be much older than the MTBC (Han and Silva 2014). *M. leprae* has clearly undergone extensive genome reduction. As a result, the *M*. *leprae* genome is much smaller than that of its mycobacterial relatives and contains a remarkably high number of pseudogenes (Gomez-Valero et al. 2007). This may indicate that if the clonal species we examined here continue to evolve clonally they may experience similar extensive genome reduction. Although, it is important to note that *M. leprae* is an obligate intracellular pathogen, which could also contribute to its patterns of gene loss.

We were able to demonstrate that most genes that are lost within the genomes of clonal bacterial species are maintained as pseudogenes. This raises the question of what causes clonal strains to maintain a higher proportion of pseudogenes than most recombining species. One possibility is that the maintenance of pseudogenes is due to the low rates of recombination within these species. It is reasonable to predict that the process of recombination that leads to the addition of genetic material into bacterial species may also be responsible for purging genetic material out of the genome. Under this model lack of HGT will lead to both a reduction in the addition of new genes and in the purging of pseudogenes. A second explanation, which we consider to be less likely, is that maintenance of pseudogenes has an adaptive effect for clonal bacteria. Pseudogene maintenance may allow them to revert back and forth from having a functional form of a protein to having a nonfunctional one and vice versa. This in turn may allow expansion of their phenotypic space under the constraints of HGT absence. It is unclear to us how such a second-order selective force would work. However, it is also possible that the maintenance of pseudogenes is due to lack of recombination, and is not directly adaptive, but that this nevertheless allows clonal species to expand their phenotypic space.

Considering the low levels of sequence variation observed for the clonal species, it is also possible that pseudogenes have simply not had enough time to be efficiently removed from their genomes. However, within this context it is, again, interesting to consider *M. leprae.* As we already mentioned *M. leprae* is an “older” relative of the MTBC that has also been evolving clonally (Monot et al. 2005; Han and Silva 2014). *M. leprae* tends to maintain a disproportionate number of pseudogenes. Indeed, the proportion of pseudogenes maintained by this bacterium is high enough to lead to it being an obvious outlier to the trend by which gene number and genome size correlate across bacteria (fig. 1). In other words, when compared with other bacterial genomes the genome of *M. leprae* is observably much too large for its number of functional genes. The “extra” DNA encoded by the *M. leprae* genome arises from over 1,000 pseudogenes that are maintained within its genome, a large fraction of which were estimated to have arisen in a massive pseudogenization event that occurred approximately 20 Ma (Gomez-Valero et al. 2007). Thus, *M. leprae* has seemingly been maintaining an extreme number of pseudogenes over a very long period of time. This implies that time may not be a dominant factor in explaining why we find a relatively high fraction of lost genes that are maintained as pseudogenes within the four examined clonal species. However, the exact causes of pseudogene maintenance within clonal bacteria need to be further studied.

Irrespective of the reasons for frequent gene loss and maintenance of pseudogenes, our results clearly demonstrate that gene loss is a dominant factor in generating genetic variation within clonal bacterial species. The variation in gene content resulting from such gene loss is therefore a potential dominant contributor to the generation of phenotypic variation between strains of such species. It is thus important that such variation be considered when trying to understand why different strains of such important pathogens as the MTBC show extensive phenotypic variation.

## Supplementary Material

Supplementary figures S1 and S2 and tables S1–S6 are available at *Genome Biology and Evolution* online (http://www.gbe.oxfordjournals.org/).

Supplementary Data
