# Supplementary material for: Gene Loss Dominates As a Source of Genetic Variation within Clonal Pathogenic Bacterial Species
Source: Genome Biol Evol. 2015 Jul 10;7(8):2173–87. doi: 10.1093/gbe/evv135 (PMC4558853; doi:10.1093/gbe/evv135)
Supplement: Supplementary Data [file supp_7_8_2173__index.html]

Gene Loss Dominates As a Source of Genetic Variation within Clonal Pathogenic Bacterial Species — Supplementary Data 

# Gene Loss Dominates As a Source of Genetic Variation within Clonal Pathogenic Bacterial Species

## Supplementary Data

files

- Supplementary Data - pdf file
