# Supplementary material for: Gene Loss Dominates As a Source of Genetic Variation within Clonal Pathogenic Bacterial Species
Source: Genome Biol Evol. 2015 Jul 10;7(8):2173–87. doi: 10.1093/gbe/evv135 (PMC4558853; doi:10.1093/gbe/evv135)
Supplement: Supplementary Data [file supp_evv135_Joined_supplementary_data.pdf]

Figure S1

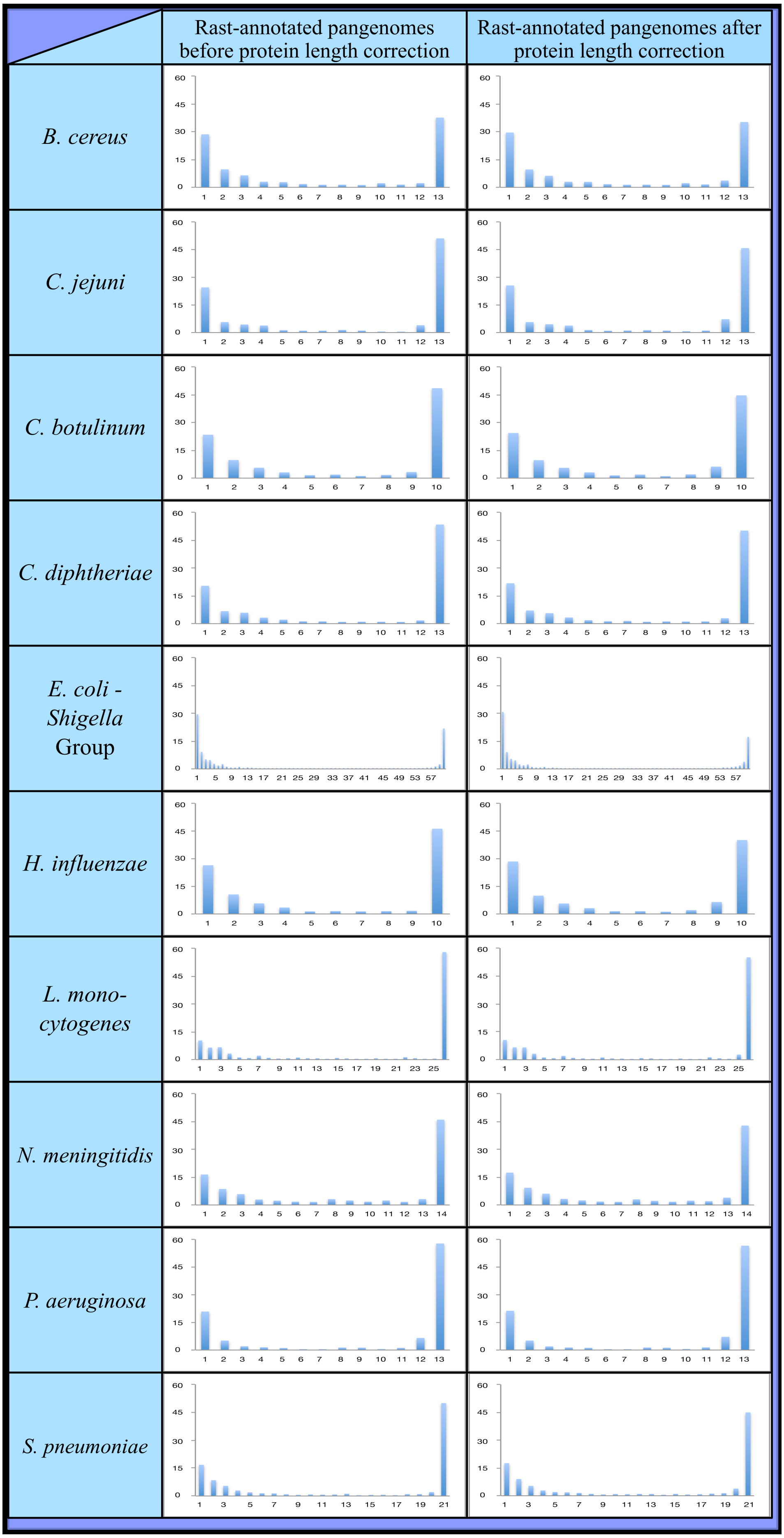

Rast-annotated pangenomes before protein length correction
Rast-annotated pangenomes after protein length correction
B. cereus
C. jejuni
C. botulinum
C. diphtheriae
E. coli - Shigella Group
H. influenzae
L. mono-cytogenes
N. meningitidis
P. aeruginosa
S. pneumoniae

Figure S2

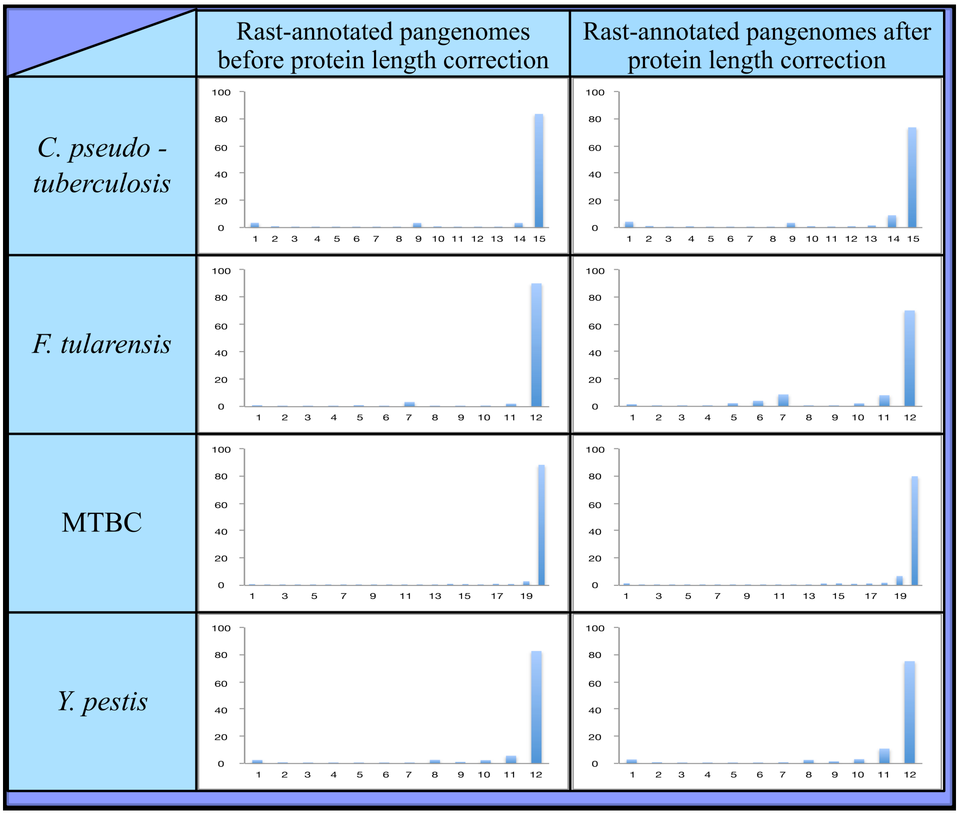
Rast-annotated pangenomes before protein length correction
Rast-annotated pangenomes after protein length correction
C. pseudo - tuberculosis
F. tularensis
MTBC
Y. pestis

Supplementary Figure legends

Figure S1. Pangenome plots of ten recombining bacterial species based on RAST annotated genomes. To generate these plots all strains within a species were annotated using the RAST annotation webserver and the pangenomes were generated as described above. Two plots are provided for each species: Left: HGT-artifact corrected pangenome plot – the protein pangenome was compared to the full DNA sequences of the strains from which it was generated. Pangenes appearing in less than 50% of strains within a species at the annotated protein level, but in more than 75% of the strains at the whole DNA level, were removed from the plot; Right: Protein length corrected pangenome plot – for each pangene within the HGT-artifact corrected pangenome lengths of all genes within the pangene group were compared to the length of the longest gene within the group. Genes, whose length was shorter than two thirds of the longest gene length, were removed from the pangene cluster and the pangene distribution was re-drawn.

Figure S2. Pangenome plots of four clonal bacterial species based on RAST annotated genomes. To generate these plots all strains within a species were annotated using the RAST annotation webserver and the pangenomes were generated as described above. Two plots are provided for each species: Left: HGT-artifact corrected pangenome plot – the protein pangenome was compared to the full DNA sequences of the strains from which it was generated. Pangenes appearing in less than 50% of strains within a species at the annotated protein level, but in more than 75% of the strains at the whole DNA level, were removed from the plot; Right: Protein length corrected pangenome plot – for each pangene within the HGT-artifact corrected pangenome lengths of all genes within the pangene group were compared to the length of the longest gene within the group. Genes, whose length was shorter than two thirds of the longest gene length, were removed from the pangene cluster and the pangene distribution was re-drawn.

Table S1. Investigated strains and the percentage of "near core" pangenes absent from each strain

Bacilllus cereus			
**Accession number**	**Strain Name**	**Number of genes[a]**	**Percentage of lost genes[b]**
NC_003909	Bacillus cereus ATCC 10987	5376	1.66
NC_004722	Bacillus cereus ATCC 14579	4998	3.30
NC_006274	Bacillus cereus E33L	4960	1.67
NC_011658	Bacillus cereus AH187	5169	0.21
NC_011725	Bacillus cereus B4264	5202	0.79
NC_011772	Bacillus cereus G9842	5245	1.22
NC_011773	Bacillus cereus AH820	5244	0.51
NC_011969	Bacillus cereus Q1	4973	0.88
NC_012472	Bacillus cereus 03BB102	5166	0.39
NC_014335	Bacillus cereus biovar anthracis str. CI	4990	0.68
NC_016771	Bacillus cereus NC7401	5169	0.21
NC_016779	Bacillus cereus F837/76	5160	1.03
NC_018491	Bacillus cereus FRI-35	4953	1.31
Campylobacter jejuni			
**Accession number**	**Strain Name**	**Number of genes**	**Percent of lost genes**
NC_003912	Campylobacter jejuni RM1221	1761	0.68
NC_008787	Campylobacter jejuni subsp. jejuni 81-176	1614	0.12
NC_009707	Campylobacter jejuni subsp. doylei 269.97	1643	8.64
NC_009839	Campylobacter jejuni subsp. jejuni 81116	1599	0.19
NC_014802	Campylobacter jejuni subsp. jejuni ICDCCJ07001	1474	10.18
NC_017279	Campylobacter jejuni subsp.	1583	0.19

	jejuni IA3902		
NC_017280	Campylobacter jejuni subsp. jejuni M1	1594	0.63
NC_017281	Campylobacter jejuni subsp. jejuni S3	1695	0.53
NC_018521	Campylobacter jejuni subsp. jejuni NCTC 11168-BN148,	1591	0.13
NC_018709	Campylobacter jejuni subsp. jejuni PT14	1576	0.25
NC_022352	Campylobacter jejuni subsp. jejuni 00-2426	1656	0.18
NC_022353	Campylobacter jejuni subsp. jejuni 00-2544	1683	0.48
NC_022529	Campylobacter jejuni 4031	1639	0.00
Clostridium botulinum			
**Accession number**	**Strain Name**	**Number of genes**	**Percentage of lost genes**
NC_009495	Clostridium botulinum A str. ATCC 3502	3385	0.65
NC_009697	Clostridium botulinum A str. ATCC 19397	3348	0.75
NC_009698	Clostridium botulinum A str. Hall	3171	1.89
NC_009699	Clostridium botulinum F str. Langeland	3436	0.41
NC_010516	Clostridium botulinum B1 str. Okra	3477	0.86
NC_010520	Clostridium botulinum A3 str. Loch Maree	3470	2.33
NC_012563	Clostridium botulinum A2 str. Kyoto	3603	0.92
NC_012658	Clostridium botulinum Ba4 str. 657	3455	3.15

NC_017297	Clostridium botulinum F str. 230613	3316	5.46
NC_017299	Clostridium botulinum H04402 065	3457	2.49
Corynebacterium diphtheriae			
**Accession number**	**Strain Name**	**Number of genes**	**Percentage of lost genes**
NC_002935	Corynebacterium diphtheriae NCTC 13129	2159	1.02
NC_016782	Corynebacterium diphtheriae 241	2091	0.57
NC_016783	Corynebacterium diphtheriae INCA 402	2090	0.57
NC_016785	Corynebacterium diphtheriae CDCE 8392	2098	0.52
NC_016786	Corynebacterium diphtheriae HC01	2100	0.43
NC_016787	Corynebacterium diphtheriae HC03	2115	0.24
NC_016788	Corynebacterium diphtheriae HC04	2106	0.33
NC_016789	Corynebacterium diphtheriae PW8	2105	1.66
NC_016790	Corynebacterium diphtheriae VA01	2067	0.10
NC_016799	Corynebacterium diphtheriae 31A	2204	0.32
NC_016800	Corynebacterium diphtheriae BH8	2203	0.68
NC_016801	Corynebacterium diphtheriae C7 (beta)	2183	0.46
NC_016802	Corynebacterium diphtheriae HC02	2104	0.52

Corynebacterium pseudotuberculosis			
**Accession number**	**Strain Name**	**Number of genes**	**Percentage of lost genes**
NC_014329	Corynebacterium pseudotuberculosis FRC41	2106	1.52
NC_016781	Corynebacterium pseudotuberculosis 3/99-5	2131	0.70
NC_016932	Corynebacterium pseudotuberculosis 316	2099	2.14
NC_017031	Corynebacterium pseudotuberculosis P54B96	2078	0.96
NC_017300	Corynebacterium pseudotuberculosis 1002	2085	0.58
NC_017301	Corynebacterium pseudotuberculosis C231	2086	0.91
NC_017303	Corynebacterium pseudotuberculosis I19	2089	0.48
NC_017305	Corynebacterium pseudotuberculosis PAT10	2073	0.87
NC_017306	Corynebacterium pseudotuberculosis 42/02-A	2046	0.98
NC_017307	Corynebacterium pseudotuberculosis CIP 52.97	2046	2.25
NC_017308	Corynebacterium pseudotuberculosis 1/06-A	1957	4.14
NC_017462	Corynebacterium pseudotuberculosis 267	2140	0.98
NC_017730	Corynebacterium pseudotuberculosis 31	2059	2.96
NC_017945	Corynebacterium pseudotuberculosis 258	2077	2.02
NC_018019	Corynebacterium pseudotuberculosis Cp162	1989	3.97

Escherichia coli-Shigella Group			
**Accession number**	**Strain Name**	**Number of genes**	**Percentage of lost genes**
NC_000913	Escherichia coli str. K-12 substr. MG1655	3659	0.90
NC_002655	Escherichia coli O157:H7 str. EDL933	4099	0.85
NC_002695	Escherichia coli O157:H7 str. Sakai	4111	1.61
NC_004431	Escherichia coli CFT073	4258	1.32
NC_007779	Escherichia coli str. K-12 substr. W3110	3686	0.79
NC_007946	Escherichia coli UTI89	4176	0.67
NC_008253	Escherichia coli 536	3999	1.28
NC_008563	Escherichia coli APEC O1	3795	4.16
NC_009800	Escherichia coli HS	3875	2.55
NC_009801	Escherichia coli E24377A	4090	1.12
NC_010468	Escherichia coli ATCC 8739	3731	1.05
NC_010498	Escherichia coli SMS-3-5	4156	0.53
NC_011353	Escherichia coli O157:H7 str. EC4115	4092	2.49
NC_011415	Escherichia coli SE11	4087	0.05
NC_011601	Escherichia coli O127:H6 str. E2348/69	3915	1.61
NC_011741	Escherichia coli IAI1	3882	0.26
NC_011742	Escherichia coli S88	3929	0.92
NC_011745	Escherichia coli ED1a	4037	2.43
NC_011748	Escherichia coli 55989	3962	0.88
NC_011750	Escherichia coli IAI39	3941	2.82
NC_011751	Escherichia coli UMN026	4157	0.72
NC_011993	Escherichia coli LF82	3911	0.56
NC_012759	Escherichia coli BW2952	3568	2.13
NC_012967	Escherichia coli B str. REL606	3665	2.21

NC_012971	Escherichia coli BL21(DE3)	3614	2.71
NC_013008	Escherichia coli O157:H7 str. TW14359	4090	1.74
NC_013353	Escherichia coli O103:H2 str. 12009	3946	1.12
NC_013361	Escherichia coli O26:H11 str. 11368	4036	1.29
NC_013364	Escherichia coli O111:H- str. 11128	3904	1.84
NC_013654	Escherichia coli SE15	3858	0.70
NC_013941	Escherichia coli O55:H7 str. CB9615	4104	0.90
NC_016902	Escherichia coli KO11FL	3991	0.38
NC_017625	Escherichia coli DH1	3676	1.03
NC_017626	Escherichia coli 042	4080	1.76
NC_017628	Escherichia coli IHE3034	4016	1.22
NC_017631	Escherichia coli ABU 83972	4081	0.71
NC_017633	Escherichia coli ETEC H10407	3925	1.45
NC_017635	Escherichia coli W	3936	0.69
NC_017641	Escherichia coli UMNK88	4321	0.88
NC_017646	Escherichia coli O7:K1 str. CE10	4183	1.20
NC_017652	Escherichia coli str. 'clone D i14'	4242	0.73
NC_017656	Escherichia coli O55:H7 str. RM12579	4085	0.56
NC_017663	Escherichia coli P12b	3721	6.21
NC_017906	Escherichia coli Xuzhou21	4025	1.61
NC_018650	Escherichia coli O104:H4 str. 2009EL-2050	4037	0.50
NC_018658	Escherichia coli O104:H4 str. 2011C-3493	4064	0.49
NC_018661	Escherichia coli O104:H4 str.	4039	0.50

	2009EL-2071		
NC_020163	Escherichia coli APEC O78	3946	0.38
NC_022364	Escherichia coli LY180	3914	1.28
NC_022370	Escherichia coli PMV-1 main	3901	1.00
NC_022648	Escherichia coli JJ1886	4066	1.03
NC_004337	Shigella flexneri 2a str. 301	3137	12.88
NC_004741	Shigella flexneri 2a str. 2457T	3131	12.94
NC_007384	Shigella sonnei Ss046	3276	8.91
NC_007606	Shigella dysenteriae Sd197	2799	23.26
NC_007613	Shigella boydii Sb227	3121	12.82
NC_008258	Shigella flexneri 5 str. 8401	3208	11.47
NC_010658	Shigella boydii CDC 3083-94	3150	14.35
NC_016822	Shigella sonnei 53G	3840	3.96
NC_017328	Shigella flexneri 2002017	3397	10.77
Francisella tularensis			
**Accession number**	**Strain Name**	**Number of genes**	**Percentage of lost genes**
NC_006570	Francisella tularensis subsp. tularensis SCHU S4	1374	0.51
NC_007880	Francisella tularensis subsp. holarctica LVS	1516	0.79
NC_008245	Francisella tularensis subsp. tularensis FSC198	1419	0.56
NC_008369	Francisella tularensis subsp. holarctica OSU18	1397	2.15
NC_009257	Francisella tularensis subsp. tularensis WY96-3418	1452	1.65
NC_009749	Francisella tularensis subsp. holarctica FTNF002-00	1370	1.53
NC_010677	Francisella tularensis subsp. mediasiatica FSC147	1286	6.07
NC_016933	Francisella tularensis TIGB03	1419	0.21
NC_016937	Francisella tularensis subsp.	1418	0.21

	tularensis TI0902		
NC_017453	Francisella tularensis subsp. tularensis NE061598	1581	0.19
NC_019537	Francisella tularensis subsp. holarctica F92	1564	1.79
NC_019551	Francisella tularensis subsp. holarctica FSC200	1318	3.26
Haemophilus influenzae			
**Accession number**	**Strain Name**	**Number of genes**	**Percentage of lost genes**
NC_000907	Haemophilus influenzae Rd KW20	1545	2.78
NC_007146	Haemophilus influenzae 86-028NP	1709	0.47
NC_009566	Haemophilus influenzae PittEE	1539	5.65
NC_009567	Haemophilus influenzae PittGG	1581	9.23
NC_014920	Haemophilus influenzae F3031	1676	2.51
NC_014922	Haemophilus influenzae F3047	1681	1.84
NC_016809	Haemophilus influenzae 10810	1765	0.34
NC_017451	Haemophilus influenzae R2866	1723	0.64
NC_017452	Haemophilus influenzae R2846	1570	0.57
NC_022356	Haemophilus influenzae KR494	1648	1.82
Listeria monocytogenes			
**Accession number**	**Strain Name**	**Number of genes**	**Percentage of lost genes**
NC_002973	Listeria monocytogenes serotype 4b str. F2365	2779	0.94
NC_003210	Listeria monocytogenes EGD-e	2818	0.18
NC_011660	Listeria monocytogenes HCC23	2917	3.36
NC_012488	Listeria monocytogenes	2729	0.55

	Clip81459		
NC_013766	Listeria monocytogenes 08-5578	2945	0.31
NC_013768	Listeria monocytogenes 08-5923	2911	0.27
NC_017529	Listeria monocytogenes L99	2874	3.24
NC_017537	Listeria monocytogenes M7	2920	3.15
NC_017544	Listeria monocytogenes 10403S	2781	0.18
NC_017545	Listeria monocytogenes J0161	2894	0.38
NC_017546	Listeria monocytogenes FSL R2-561	2861	0.28
NC_017547	Listeria monocytogenes Finland 1998	2742	0.55
NC_018584	Listeria monocytogenes ATCC 19117	2816	0.11
NC_018585	Listeria monocytogenes SLCC2378	2824	0.14
NC_018586	Listeria monocytogenes SLCC2540	2856	0.11
NC_018587	Listeria monocytogenes SLCC2755	2828	0.11
NC_018588	Listeria monocytogenes SLCC2372	2885	0.10
NC_018589	Listeria monocytogenes SLCC2479	2882	0.10
NC_018590	Listeria monocytogenes SLCC2376	2731	2.71
NC_018591	Listeria monocytogenes serotype 7 str. SLCC2482	2826	0.04
NC_018592	Listeria monocytogenes SLCC5850	2833	0.11
NC_018593	Listeria monocytogenes	2786	0.22

	SLCC7179		
NC_018642	Listeria monocytogenes L312	2778	0.11
NC_019556	Listeria monocytogenes serotype 4b str. LL195	2797	0.86
NC_021829	Listeria monocytogenes J1816	2744	3.79
NC_022568	Listeria monocytogenes EGD	2820	0.11
Mycobacterium tuberculosis Complex			
**Accession number**	**Strain Name**	**Number of genes**	**Percentage of lost genes**
NC_002755	Mycobacterium tuberculosis CDC1551	3933	2.85
NC_002945	Mycobacterium bovis AF2122/97	3665	0.19
NC_008769	Mycobacterium bovis BCG str. Pasteur 1173P2	3642	0.69
NC_009525	Mycobacterium tuberculosis H37Ra	3747	0.32
NC_009565	Mycobacterium tuberculosis F11	3662	0.96
NC_012207	Mycobacterium bovis BCG str. Tokyo 172	3661	0.25
NC_012943	Mycobacterium tuberculosis KZN 1435	3781	0.11
NC_015758	Mycobacterium africanum GM041182	3578	2.07
NC_016768	Mycobacterium tuberculosis KZN 4207	3724	0.24
NC_016804	Mycobacterium bovis BCG str. Mexico	3667	0.57
NC_016934	Mycobacterium tuberculosis UT205	3602	1.92
NC_017522	Mycobacterium tuberculosis CCDC5180	3353	9.39

NC_017523	Mycobacterium tuberculosis CCDC5079	3404	9.81
NC_017524	Mycobacterium tuberculosis CTRI-2	3672	0.35
NC_018078	Mycobacterium tuberculosis KZN 605	3727	0.72
NC_018143	Mycobacterium tuberculosis H37Rv	3826	0.16
NC_020089	Mycobacterium tuberculosis 7199-99 complete genome	3719	0.38
NC_020245	Mycobacterium bovis BCG str. Korea 1168P	3826	0.73
NC_020559	Mycobacterium tuberculosis str. Erdman = ATCC 35801 DNA	3958	1.87
NC_022350	Mycobacterium tuberculosis str. Haarlem	3769	0.61
Neisseria meningitidis			
**Accession number**	**Strain Name**	**Number of genes**	**Percentage of lost genes**
NC_003112	Neisseria meningitidis MC58	1706	2.17
NC_003116	Neisseria meningitidis Z2491	1723	1.80
NC_008767	Neisseria meningitidis FAM18	1708	1.76
NC_010120	Neisseria meningitidis 053442	1775	1.24
NC_013016	Neisseria meningitidis alpha14	1737	2.71
NC_017501	Neisseria meningitidis 8013	1735	0.81
NC_017505	Neisseria meningitidis alpha710	1804	1.44
NC_017512	Neisseria meningitidis WUE 2594	1774	2.25
NC_017513	Neisseria meningitidis G2136	1713	0.99
NC_017514	Neisseria meningitidis M01-240149	1712	0.58

NC_017515	Neisseria meningitidis M04-240196	1720	0.81
NC_017516	Neisseria meningitidis H44/76	1721	0.76
NC_017517	Neisseria meningitidis M01-240355	1742	0.40
NC_017518	Neisseria meningitidis NZ-05/33	1708	0.88
Pseudomonas aeruginosa			
**Accession number**	**Strain Name**	**Number of genes**	**Percentage of lost genes**
NC_002516	Pseudomonas aeruginosa PAO1	5407	0.94
NC_008463	Pseudomonas aeruginosa UCBPP-PA14	5685	0.39
NC_009656	Pseudomonas aeruginosa PA7 chromosome	5984	6.99
NC_011770	Pseudomonas aeruginosa LESB58	5697	1.14
NC_017548	Pseudomonas aeruginosa M18	5511	0.31
NC_017549	Pseudomonas aeruginosa NCGM2.S1	6043	0.17
NC_018080	Pseudomonas aeruginosa DK2	5687	1.58
NC_020912	Pseudomonas aeruginosa B136-33,	5624	0.14
NC_021577	Pseudomonas aeruginosa RP73	5578	1.20
NC_022808	Pseudomonas aeruginosa PA1	5570	1.56
NC_023019	Pseudomonas aeruginosa MTB-1	5863	0.22
NC_023066	Pseudomonas aeruginosa LES431	5763	0.49
NC_023149	Pseudomonas aeruginosa SCV20265	6025	0.13
Streptococcus pneumoniae			

**Accession number**	**Strain Name**	**Number of genes**	**Percentage of lost genes**
NC_003028	Streptococcus pneumoniae TIGR4	1942	1.18
NC_003098	Streptococcus pneumoniae R6	1690	2.19
NC_008533	Streptococcus pneumoniae D39	1777	0.96
NC_010380	Streptococcus pneumoniae Hungary19A-6	1949	2.36
NC_010582	Streptococcus pneumoniae CGSP14	1941	0.93
NC_011072	Streptococcus pneumoniae G54	1901	0.47
NC_011900	Streptococcus pneumoniae ATCC 700669	1839	1.25
NC_012466	Streptococcus pneumoniae JJA	1882	1.01
NC_012467	Streptococcus pneumoniae P1031	1840	1.03
NC_012468	Streptococcus pneumoniae 70585	1932	0.52
NC_012469	Streptococcus pneumoniae Taiwan19F-14	1847	0.65
NC_014251	Streptococcus pneumoniae TCH8431/19A	1997	0.15
NC_014494	Streptococcus pneumoniae AP200	1981	0.66
NC_014498	Streptococcus pneumoniae 670-6B	2038	0.44
NC_017591	Streptococcus pneumoniae INV104	1717	2.33
NC_017592	Streptococcus pneumoniae OXC141	1708	2.17
NC_017593	Streptococcus pneumoniae INV200	1774	1.01
NC_017769	Streptococcus pneumoniae	1909	0.52

	ST556		
NC_018594	Streptococcus pneumoniae SPNA45	1773	5.08
NC_018630	Streptococcus pneumoniae gamPNI0373	1881	0.64
NC_022655	Streptococcus pneumoniae A026 genome	1762	2.27
Yersinia pestis			
**Accession number**	**Strain Name**	**Number of genes**	**Percentage of lost genes**
NC_003143	Yersinia pestis CO92	3459	1.36
NC_004088	Yersinia pestis KIM 10	3636	0.83
NC_005810	Yersinia pestis biovar Microtus str. 91001	3593	2.00
NC_008149	Yersinia pestis Nepal516	3633	0.00
NC_008150	Yersinia pestis Antiqua	3734	0.37
NC_009381	Yersinia pestis Pestoides F	3563	1.77
NC_010159	Yersinia pestis Angola	3467	6.86
NC_014029	Yersinia pestis Z176003	3250	1.11
NC_017154	Yersinia pestis D106004	3326	0.54
NC_017160	Yersinia pestis D182038	3315	0.94
NC_017168	Yersinia pestis A1122	3701	0.30
NC_017265	Yersinia pestis biovar Medievalis str. Harbin 35	3851	2.83

[a]Number of genes from each strain used for the construction of pangenomes (see Materials and Methods)

[b]Percent of “near core” gene loss. Calculated as 100*L/T, where L is the number of “near core” core pangenes that are absent from the genome and T is the total number of genes present within that genome, that were used to construct the species pangenome.

Table S2. List of bacterial strains removed from our analysis

Campylobacter jejuni	
**Accession number**	**Strain Name**
NC_002163	Campylobacter jejuni subsp. jejuni NCTC 11168 = ATCC 700819
NC_022351	Campylobacter jejuni subsp. jejuni 00-2538
NC_022362	Campylobacter jejuni subsp. jejuni 00-2425
NC_021834	Campylobacter jejuni 32488
Clostridium botulinum	
**Accession number**	**Strain Name**
NC_010674	Clostridium botulinum B str. Eklund 17B
NC_010723	Clostridium botulinum E3 str. Alaska E43
NC_015425	Clostridium botulinum BKT015925
***Escherichia coli-Shigella* Group**	
**Accession number**	**Strain Name**
NC_017644	Escherichia coli NA114
NC_017632	Escherichia coli UM146
NC_017634	Escherichia coli O83:H1 str. NRG 857C
NC_022912	Shigella dysenteriae 1617
NC_020518	Escherichia coli str. K-12 substr. MDS42 DNA
NC_010473	Escherichia coli str. K-12 substr. DH10B
NC_017651	Escherichia coli str. 'clone D i2'
Listeria monocytogenes	
**Accession number**	**Strain Name**
NC_017728	Listeria monocytogenes 07PF0776
NC_020557	Listeria monocytogenes La111
NC_020558	Listeria monocytogenes N53-1
NC_021830	Listeria monocytogenes J1-220
***Mycobacterium Tuberculosis* Cluster**	
**Accession number**	**Strain Name**
NC_017026	Mycobacterium tuberculosis RGTB327
NC_017528	Mycobacterium tuberculosis RGTB423

NC_021054	Mycobacterium tuberculosis str. Beijing/NITR203
NC_021192	Mycobacterium tuberculosis str. Haarlem/NITR202
NC_021193	Mycobacterium tuberculosis CAS/NITR204
NC_021194	Mycobacterium tuberculosis EAI5/NITR206
Pseudomonas aeruginosa	
**Accession number**	**Strain Name**
NC_022360	Pseudomonas aeruginosa c7447m
NC_022806	Pseudomonas aeruginosa PA1R
NC_022594	Pseudomonas aeruginosa PAO1-VE13
NC_022591	Pseudomonas aeruginosa PAO1-VE2
NC_022361	Pseudomonas aeruginosa PAO581

Table S3. Summary of recombination analyses by PHIpack software for 14 analyzed species

**Species**[a]	**Number of tested genes**	**Recombinant genes (%)**	**Non-recombinant genes (%)**	**Non-informative genes (%)**
B. cereus	1171	59.61	40.39	0.00
C. jejuni	428	33.18	66.82	0.00
C. botulinum	943	29.16	70.73	0.11
C. diphteriae	780	45.51	52.82	1.67
*C. pseudotuberculosis**	660	0.15	96.21	3.64
E. coli-Shigella	546	78.94	21.06	0.00
*F. tularensis**	467	0.00	58.24	41.76
H. influenzae	394	67.01	32.74	0.25
L. monocytogenes	549	45.90	54.10	0.00
MTBC*	1279	0.00	2.42	97.58
N. meningitidis	550	73.27	25.45	1.27
P. aeruginosa	2039	16.04	82.54	1.42
S. pneumoniae	502	31.87	64.74	3.39
*Y. pestis**	1067	0.00	0.00	100.00

[a]Clonal species are marked with an *asterisk*

Table S4. Summary of pangene frequencies within the protein pangenomes based on the NCBI annotations of the 14 studied species (prior to HGT correction)

**Species**[a]	**Pangenome size**[b]	**"Rare" pangenes**[c]	**"Near Core" pangenes**[d]	**"Core" pangenes**[e]
B.cereus	10116	47.87	5.21	33.17
C. jejuni	2551	34.42	12.35	42.06
C. botulinum	5632	41.10	9.34	41.87
C. diphtheriae	3292	32.75	3.77	51.00
*C.pseudotuberculosis**	2577	16.14	14.40	60.03
E. coli-Shigella	12282	68.02	13.02	12.43
*F. tularensis**	1781	13.53	10.61	57.50
H. influenzae	2782	42.45	12.47	37.67
L. monocytogenes	4379	34.64	6.42	51.56
*MTBC**	4490	15.70	14.30	63.74
N. meningitidis	2717	35.04	7.21	47.55
P. aeruginosa	9029	36.49	8.34	48.84
S. pneumoniae	3327	41.66	8.48	37.57
*Y. pestis**	4583	18.94	11.78	55.27

[a]Clonal species are marked with an *asterisk*

[b]Number of pangenes (orthologous gene clusters) within pangenome

[c]% of pangenes that are found in less than 25% of strains of a species

[d]% of pangenes that are found in over 75% of strains of a species, but are not found in all strains

[e]% of pangenes found in all strains of a species

Table S5. Correlations between aAAI[a] and Percentage of genes lost[b] in the different bacterial species

**Species**[c]	**Number of genomes**	**Spearman Rho**	***P*-value**
B.cereus	13	-0.61	0.036
C. jejuni	13	-0.36	0.208
C. botulinum	10	-0.48	0.150
C. diphtheriae	13	-0.58	0.040
*C.pseudotuberculosis**	15	-0.70	0.009
E. coli-Shigella	60	-0.12	0.363
*F. tularensis**	12	-0.77	0.010
H. influenzae	10	-0.22	0.496
L. monocytogenes	26	-0.32	0.110
MTBC*	20	-0.47	0.039
N. meningitidis	14	-0.70	0.012
P. aeruginosa	13	0.19	0.503
S. pneumoniae	21	0.14	0.522
*Y. pestis**	12	-0.71	0.018

[a]Denotes for each strain within each species the average AAI for its pairwise comparisons to all other strains within that species
[b]For each strain we calculated the percentage of genes that were clearly lost from that genome as 100*L/T, where L is the number of "near core" core pangenes that are absent from the genome and T is the total number of genes present within that genome, that were used to construct the species pangenome
[c]Clonal species are marked with an *asterisk*

Table S6. Summary of pangene frequencies within the RAST annotated, length-corrected protein pangenomes of the 14 studied species

**Species**[a]	**Pangenome size**[b]	**"Rare" pangenes**[c]	**"Near Core" pangenes**[d]	**"Core" pangenes**[e]
B.cereus	9589	45.3	5.3	35.2
C. jejuni	2568	35.7	8.3	45.8
C. botulinum	5499	39.6	8.2	44.6
C. diphtheriae	3309	34.5	4.1	50.1
*C.pseudotuberculosis**	2259	6.8	11.7	73.7
E. coli-Shigella	11280	62.9	12.2	17.3
*F. tularensis**	1720	2.6	10.3	70
H. influenzae	2893	44.0	8.5	40.1
L. monocytogenes	4172	31.3	5.9	55
MTBC*	4124	2.8	11.5	79.8
N. meningitidis	3284	36	8.3	42.8
P. aeruginosa	8140	28.4	8.6	56.6
S. pneumoniae	3021	36.8	7.3	44.9
*Y. pestis**	4228	4.3	14.1	75.2

[a]Clonal species are marked with an *asterisk*

[b]Number of pangenes (orthologous gene clusters) within pangenome

[c]% of pangenes that are found in less than 25% of strains of a species

[d]% of pangenes that are found in over 75% of strains of a species, but are not found in all strains

[e]% of pangenes found in all strains of a species
